# Supplementary material for: A FIJI macro for quantifying pattern in extracellular matrix
Source: Life Sci Alliance. 2021 Jan 27;4(3):e202000880. doi: 10.26508/lsa.202000880 (PMC7898596; doi:10.26508/lsa.202000880)
Supplement: Supplementary file 1 [file LSA-2020-00880_Supplemental_Data_1.docx]

TWOMBLI DOCUMENTATION

**READ ALL THE WAY THROUGH BEFORE STARTING THE TUTORIAL – IT WILL MAKE THINGS EASIER!**

**Written by Esther Wershof 21-11-19**

[1. Introduction to TWOMBLI 1](#_Toc56687911)

[Installation and dependencies 2](#_Toc56687912)

[File organisation 2](#_Toc56687913)

[2. Tutorial 3](#_Toc56687914)

[Step 0-2: File organisation and Checking image quality 3](#_Toc56687915)

[Step 3: Arranging files 6](#_Toc56687916)

[Running the macro 6](#_Toc56687917)

[Choosing parameters (steps 4-9) 6](#_Toc56687918)

[Step 4: Adjusting contrast 7](#_Toc56687919)

[Step 5: Choosing line width(s) for ridge detection and minimum branch length 8](#_Toc56687920)

[Step 6: Choosing curvature window(s) 9](#_Toc56687921)

[Step 7: Optional gap analysis 11](#_Toc56687922)

[Step 8: Thresholding for HDM 11](#_Toc56687923)

[Step 9: Recap of chosen parameters 12](#_Toc56687924)

[Step 10: Choosing directories 13](#_Toc56687925)

[Step 11: Ridge detection 13](#_Toc56687926)

[Step 12: AnaMorf & Alignment 13](#_Toc56687927)

[Step 13: HDM 13](#_Toc56687928)

[3. Running TWOMBLI 13](#_Toc56687929)

[Using a pre-existing parameter file 14](#_Toc56687930)

[Next steps 14](#_Toc56687931)

[Gap analysis 14](#_Toc56687932)

[Selecting regions of interest 16](#_Toc56687933)

[4. Quickstart guide 18](#_Toc56687934)

[5. Trouble shooting 18](#_Toc56687935)

# Introduction to TWOMBLI

Diverse extracellular matrix patterns are observed in both normal and pathological tissue. TWOMBLI stands for The Workflow Of Matrix BioLogy Informatics. The aim of TWOMBLI is to quantify matrix patterns. The name TWOMBLI is also a nod to the American artist Cy Twombly whose works are full of diverse marks and patterning.

TWOMBLI currently consists of an ImageJ macro run in Fiji, together with user documentation and a walkthrough example and comprises three stages:

Prechecks (steps 0-3) **– these steps are carried out manually, before the macro is run**

Choosing parameter values (steps 4-9) – filtering out unsuitable images and appropriately thresholding images

Deriving matrix metrics (steps 10-14) – quantifying the matrix patterns

## Installation and dependencies

TWOMBLI can be downloaded from <https://github.com/wershofe/TWOMBLI>. Download the entire directory and **unzip the folder and copy it to a convenient directory eg Desktop.**

The TWOMBLI macro has a number of dependencies. If you have not already done so please install and update Fiji and the plugins AnaMorf, Ridge Detection and BIOP (optional for gap analysis).

Installing Fiji: <https://imagej.net/Fiji/Downloads>

Installing AnaMorf, Ridge Detection and OrientationJ (for alignment): Open Fiji -> Click help -> Update -> Manage update sites -> Make sure “Biomedgroup”, “CALM” and “BIG-EPFL” are checked then click Close -> Apply changes -> Restart Fiji when instructed.

Installing BIOP (optional for gap analysis): Open Fiji -> Click help -> Update -> Manage update sites -> Add update site -> copy and paste <https://biop.epfl.ch/Fiji-Update/> into the URL heading -> Make sure it is checked -> Close -> Apply changes -> Restart Fiji when instructed.

## File organisation

The TWOMBLI folder is organised as shown in Figure 1.

NB It may be necessary to create empty folders for “HDM” etc. so that the folder structure matches what is shown in Figure 1.

All images will go inside the “Original files” folder. Images are then subdivided into Eligible and Ineligible folders. From the Eligible folder, several images will be copied to the “TestSet/TestSetInput” folder.

The output of the pipeline creates a csv file in the TWOMBLI folder called “TwombliResults.csv” with all the metrics listed per image. More detailed output is given in the subdirectories e.g. Masks of the original images together with an AnaMorf folder containing a csv with metrics (in the “Masks” folder) and copies of the images that have been thresholded for high-density matrix (HDM) together with a csv file outputting these values (in the “HDM” directory).

There are three essential files in the Programs folder:

- Twombli_v1.ijm macro – source code for TWOMBLI
- Parameterfile.txt – stores chosen parameters. You have the option to directly edit this file and skip directly ahead to Step 9
- AnaMorfProperties.xml – Stores AnaMorf properties – we advise leaving this alone.


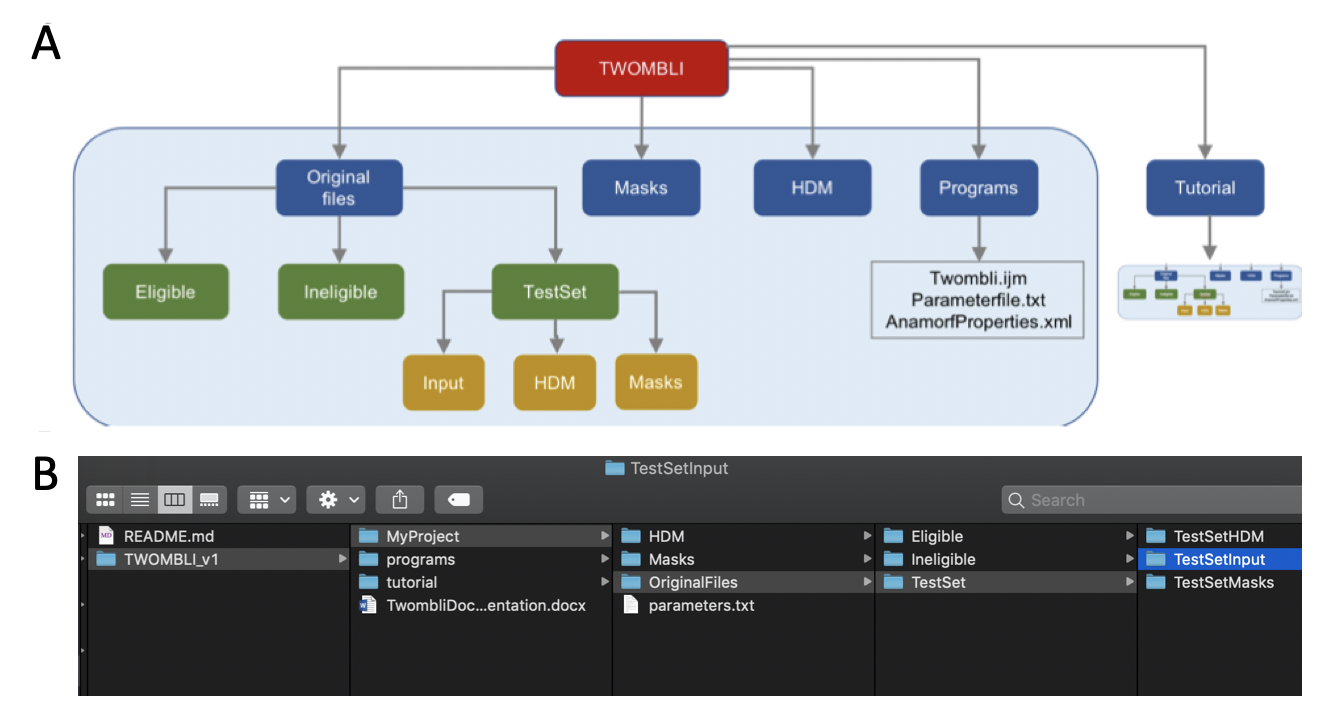


Figure 1: TWOMBLI file organisation. (A) The Tutorial folder contains an exact copy of everything in the light blue rectangle. (B) Screenshot of how folders should be organised.

# Tutorial

The purpose of the tutorial is to get a feel for what images are eligible and ineligible and suitable parameters. All tutorial images are described in Figure 2 and Table 1. The images contain breast cancer tumour cores with diameter approximately $700\mu m$, which have been stained for collagen. When you try with your own images, the procedure is the same as the tutorial, just put your files in the A new sub-folder folder rather than the Tutorial sub-folder (see the “MyProject” example in Figure 1B). The tutorial contains all the output files as an example. **When you are ready to start the tutorial, delete all the files in tutorial/HDM, tutorial/Masks, tutorial/OriginalFiles/TestSetHDM & tutorial/OriginalFiles/TestSetMasks.**

**PLEASE READ THROUGH THE WHOLE OF THIS DOCUMENTATION FILE BEFORE ATTEMPTING TO DO THE TUTORIAL**

## Step 0-2: File organisation and Checking image quality

This first step of checking and organising images into the correct folders is manual.

Click on the Tutorial/OriginalFiles folder. There are three folders: “Eligible” “Ineligible”, “TestSet”.

Images must be checked for suitability for matrix analysis. Open all images in Fiji (other image viewers can be used if preferred) either by dragging and dropping or File->Import->Image Sequence. Eligible images must satisfy the following criteria:

1. Resolution of at least 1 pixel = 1 micron (2L_7D_lowRes fails this). **All images in the tutorial have 1 pixel = 0.8 microns so satisfy this criterion.** You can sometimes check the resolution of an image open in Fiji by clicking Image->Show info. Note in the tutorial, this information is not included.
2. Sample is in focus. The matrix fibres that can be resolved will be determined by the numerical aperture of the lens used to acquire the image. More information can be found [here](https://eur03.safelinks.protection.outlook.com/?url=https%3A%2F%2Fwww.microscopyu.com%2Fmicroscopy-basics%2Fnumerical-aperture&data=02%7C01%7C%7Ca87820e6675041b7226a08d71504b3e8%7C4eed7807ebad415aa7a99170947f4eae%7C0%7C0%7C637000982252281061&sdata=eeCJaYQq41p7fUDiTqHpoKZ9ixJhBJKbUS5d0MvVxvA%3D&reserved=0) (2L_7D_blurred fails this).
3. Sample needs contain clear matrix fibre structure that has not been damaged or contaminated. By contaminated, we mean containing areas of non-fibrous tissue (10L_5D, 7L_3D, 6L_9D fail this).

Figure 2 and Table 1 detail this selection process.

Table 1: Guidance for choosing eligibility

| 2L_7D | Some adipose (yellow) tissue but good fibre structure | ELIGIBLE |
| --- | --- | --- |
| 2L_7D_blurred | Out of focus | INELIGIBLE |
| 2L_7D_lowRes | Low resolution | INELIGIBLE |
| 2L_2D | Some non-collagen tissue but good fibre structure | ELIGIBLE |
| 2L_3D | Some adipose (yellow) tissue but good fibre structure | ELIGIBLE |
| 2L_4D | Some non-collagen tissue but good fibre structure | ELIGIBLE |
| 10L_5D | Damaged/contaminated/not enough fibre | INELIGIBLE |
| 7L_3D | Borderline: OK fibre on left, contamination/damage on right | INELIGIBLE |
| 6L_9D | Damaged/contaminated/not enough fibre | INELIGIBLE |
| 2L_8D | Borderline: good fibre on left, absence of tissue on right | ELIGIBLE |

**NB for some images, it may be necessary to crop them down if the files are too big (more than 3000x3000 pixels) or if they contain large parts of the image that would render them ineligible eg edge artefacts.**


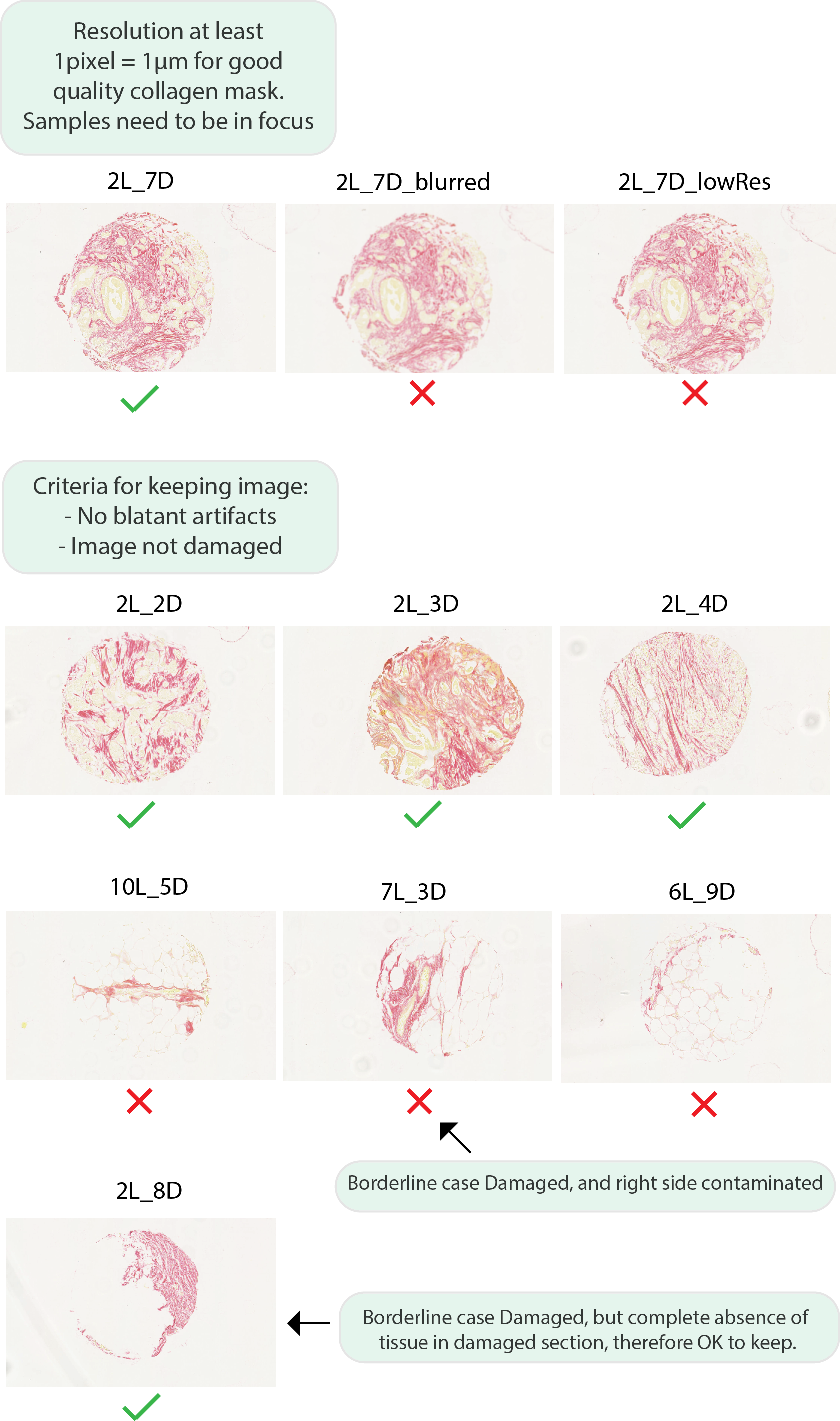


Figure 2: Choosing eligible images

## Step 3: Arranging files

This is the last manual step. If you have previously opened images in Fiji, they will close automatically. If you have used an alternative image viewer, close the images.

Move the images that fail any of the suitability criteria into the **Tutorial/OriginalFiles/Ineligible** folder. Move the remaining images into the **Tutorial/OriginalFiles/Eligible** folder.

From the images in the Eligible folder, select three to **copy** into the **Tutorial/OriginalFiles/TestSet/TestSetInput** folder. These can be chosen at random, ideally to be fair representation of the types of images to be processed. For this walkthrough however, select 2L_3D, 2L_4D, 2L_7D to copy to TestSetInput.

The images should be then organised as shown in Table 2.

Table 2: Eligibility of example images

| **Ineligible** | **Eligible** | **TestSet** |
| --- | --- | --- |
| 6L_9D | 2L_2D | 2L_3D |
| 7L_3D | 2L_3D | 2L_4D |
| 10L_5D | 2L_4D | 2L_7D |
| 2L_7D_blurred | 2L_7D |  |
| 2L_7D_lowRes | 2L_8D |  |

## Running the macro

In Fiji, click Plugins -> Macros -> Run -> Select twombli.ijm in twombli/Programs

Instructions will appear in the Log, you will be prompted to maximise the Log window so that you can read the instructions easily. The macro will check that you have completed steps 0-3 before proceeding and this is displayed in the Log.

You will first be asked if you have a pre-existing parameter file you wish to load. If you have already worked out suitable parameter values for a set of images, this step enables you to skip steps 0-9 and directly select a file with all the predefined parameter values. For now, select “No”. The log will then ask if you have completed steps 1-3.

## Choosing parameters (steps 4-9)

In steps 4-9, you will be asked to choose values for the following parameters:

Table 3: parameters to choose

| Contrast Saturation | Saturates this percentage of pixels | Step 4 |
| --- | --- | --- |
| Line Width | Ridge detection will trace a mask looking for matrix fibre bundles of approximately this width. | Step 5 |
| Curvature Window | The length over which the change in angle will be measured | Step 6 |
| Minimum Branch Length | Branches on mask below this length will be ignored (set by default to 10% of Curvature Window) by AnaMorf | Step 6 |
| Minimum gap diameter | For optional gap analysis | Step 7 |
| Maximum Display HDM | Pixel intensities above this value will be ignored. | Step 8 |

**NB the first time you run the tutorial, you may want to accept all of the default parameter values (just click “OK” or “yes” through steps 4-8) to see how the end result quickly.**

**NB the value you choose for each parameter is fixed for each image.**

## Step 4: Adjusting contrast

You will be prompted to open the testSet images (Tutorial/ OriginalFiles/TestSet/TestSetInput), an output file for TestSet Masks (Tutorial/OriginalFiles/TestSet/TestSetMasks) and an output file for TestSet HDM images (Tutorial/OriginalFiles/TestSet/TestSetHDM).

The test images will automatically open and have their contrast enhanced to a default saturation value of 0.35. You will be asked to decide if the images are now sufficiently sharp. In most cases this level will be satisfactory. If you think the image needs further enhancing, click “no”. You will then be asked to enter a saturation value between 0 and 1. By trial and error, try a different saturation value. You will be asked able to try different values until you are happy with the contrast of the image (Figure 3). **As with all of the parameters, the chosen value has to be the same for all images. Therefore, it is important to choose a value that works best across all the TestSet images.**


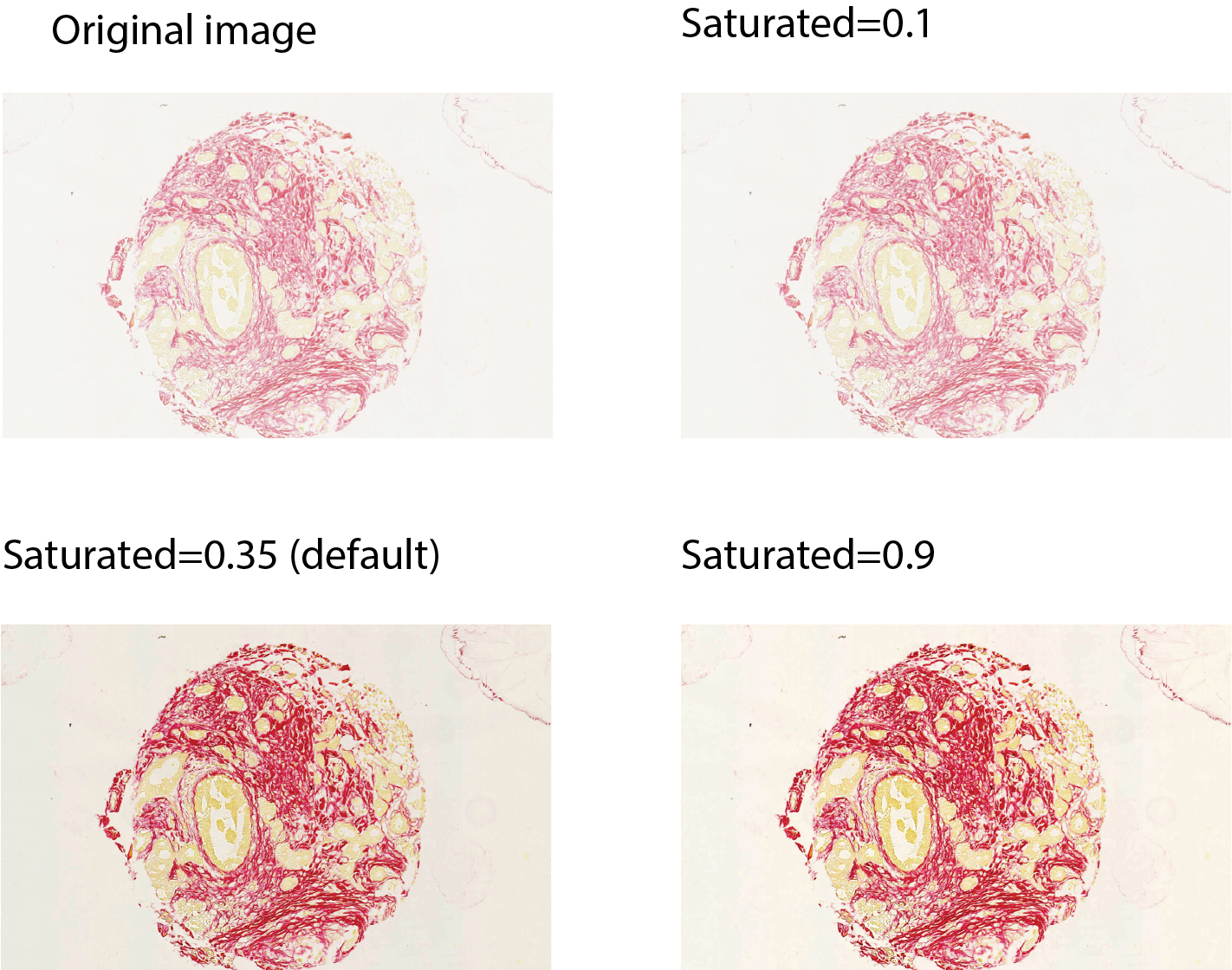


Figure 3: Thresholding for image contrast

## Step 5: Choosing line width(s) for ridge detection and minimum branch length

Ridge detection is able to identify curvilinear structures. We use it to identify the main structure of matrix fibres in the images (more information can be found [here](https://imagej.net/Ridge_Detection)). You will be asked if the matrix fibres are dark on a light background. In the case of the tutorial the answer is yes.

You will then be instructed to select proposed minimum and maximum line widths for ridge detection. **For the tutorial we recommend a minimum test value of 5 and a maximum test value of 20.** TWOMBLI will then generate masks for line width between these values at increments of 5. Open the generated masks in **TWOMBLI/Tutorial/OriginalFiles/TestSetMasks** and compare different line widths. **TestingMultipleLineWidths.csv** will tell you the total length of fibres picked up at each line width (i.e. a higher value indicates picking up more fibres, but potentially more noise). Find a line width that generates masks with the most detailed fibres, without picking up background noise. **For the tutorial, we think a line width of 5 gives the best mask.** You will have the chance to repeat this step until you are happy with the line width.

In most cases we recommend setting the minimum and maximum values to be the same, thereby evaluating a single line width. The user may wish to derive a mask with a range of line widths, in the event that the images consist of both thick and thin fibres. In this case they can set the minimum and maximum to different values. TWOMBLI will then derive a mask which is a composite of fibres at every line width between the minimum and maximum values. For example, if she selects minimum and maximum widths of 5 and 10 then ridge detection will be run for line widths of {5, 6, 7, 8, 9, 10}. This can take longer to run. Masks will be generated and saved in **TWOMBLI/Tutorial/OriginalFiles/TestSetMasks**.

Once you have chosen minimum and maximum line widths, additional masks will be generated with varying minimum branch length. This determines the pruning of short branches to avoid spurious edges/artefacts. These masks will also appear in **TWOMBLI/Tutorial/OriginalFiles/TestSetMasks** with “MIN_BRANCH_LENGTH” in the titles. In the case of the test images a minimum branch length of 5 is appropriate but if the user may find a larger minimum branch length generates a more faithful mask in their images. The user will then be prompted to enter a minimum branch length.


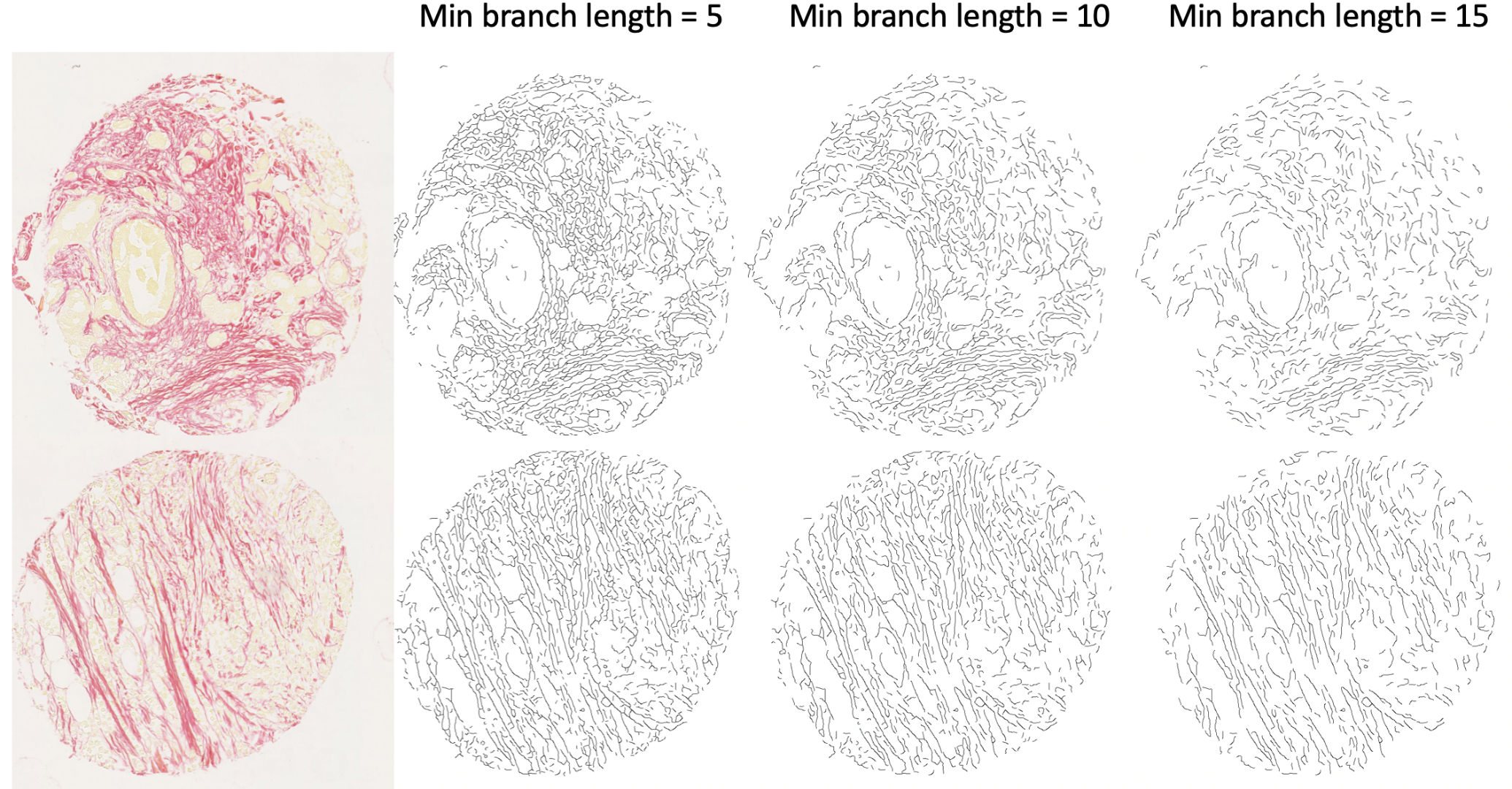


Figure 4: Choosing minimum branch length. Masks will automatically be generated for test images, pruning branches below a range of threshold values.

## Step 6: Choosing curvature window(s)

The log will prompt you to run Ridge Detection for all of the TestSet images, creating a mask. Using the Straight Line tool in Fiji, measure typical length of curves on the masks without branching or significant changes in direction. The method for measuring these lines with the Straight Line tool is described in Figure . Curvature window should be chosen to be about half this length. This can be quite a subjective call, making it advisable to try a few different curvature windows. For the walkthrough examples we recommend a curvature window of 40. The user may wish to evaluate a range of curvature windows, with smaller windows picking up waviness of individual fibres and larger windows measuring curvature at a more global scale. The user will be asked to specify a minimum and maximum curvature window. If the user is happy with a single window, they can set these two values to be the same. If the user wishes to measure multiple curvature windows, they can set the minimum and maximum to different values. TWOMBLI will then evaluate the curvature for windows of increments of 10 between the minimum and maximum. For example, if she selects minimum and maximum curvature windows of 20 and 50 then curvature will be evaluated at windows of {20, 30, 40, 50}. Each window will be given as a separate column in the output results file. This can take longer to run. **As with all of the parameters, the chosen value(s) has to be the same for all images. Therefore, it is important to choose a value that works best across all the TestSet images.**


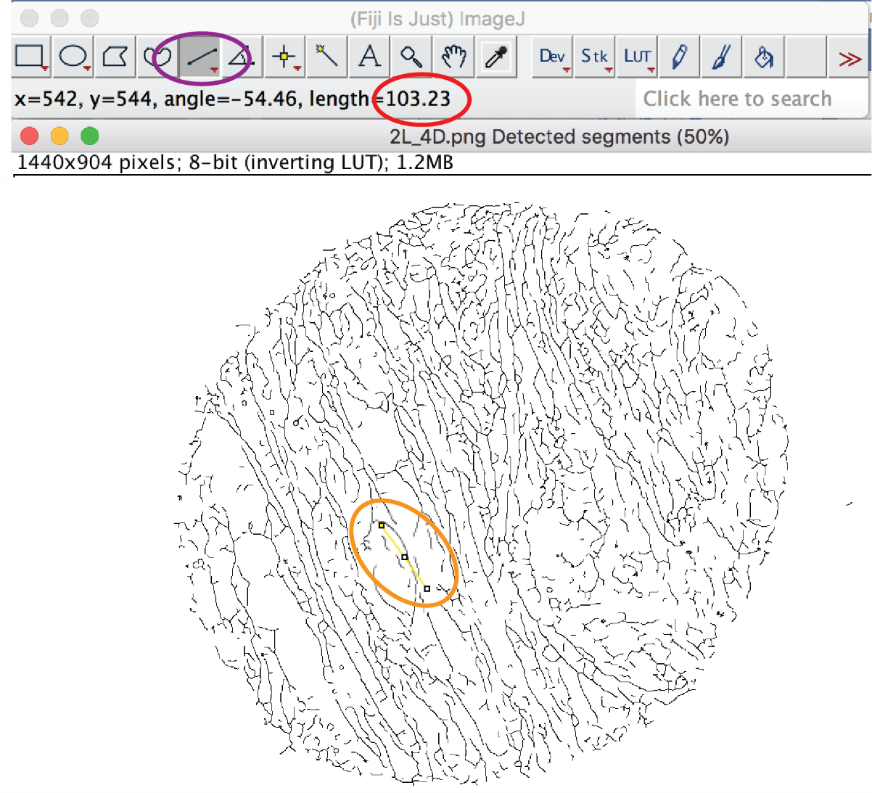


Figure 5: Measuring curvature window. Click on the straight line tool (circled in purple) and draw lines (example circled in orange) to establish an approximate length of lines which are largely straight, without branching. The length of the line may appear automatically (circled in red). Otherwise click Analyze->Measure.


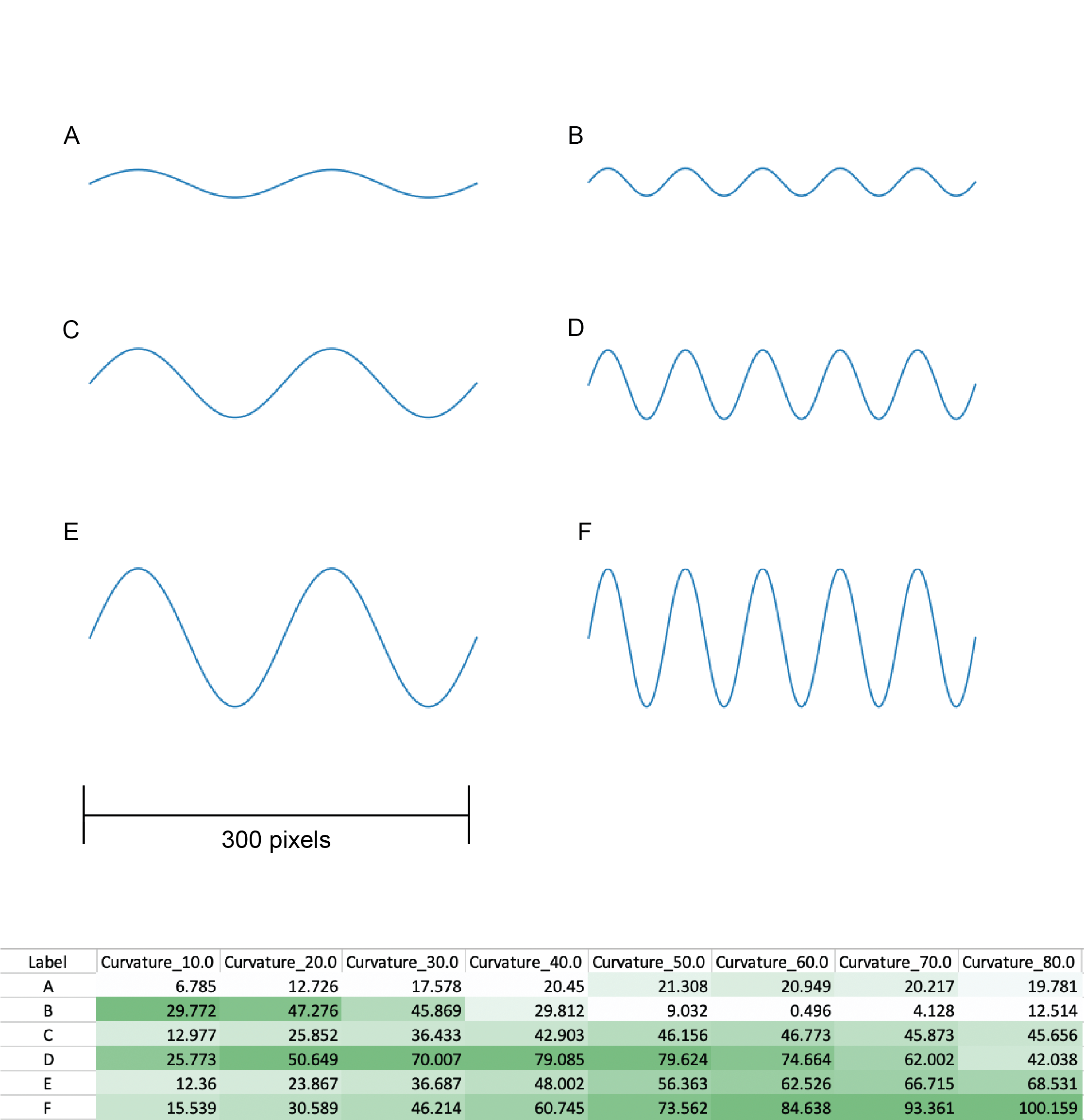


Figure 6: Applying multiple curvature windows. Curves with different periodicity and amplitude are evaluated for different curvature windows. For example, the curve in (B) has higher curvature for a lower curvature window and lower curvature for a higher curvature window, capturing it’s structure.

## Step 7: Optional gap analysis

You will be asked if you want to include gap analysis. For the tutorial, we recommend clicking “No”.

## Step 8: Thresholding for HDM

The testSet images will automatically reopen and be converted to 8-bit. The Brightness and Contrast window will automatically open. Adjust the Maximum slide bar (the second bar from the top) to remove the fainter parts of the image until only matrix remains (see Figure 4). Do not worry if you can no longer see some of the fainter matrix fibres. Once you are happy with a value that works for all TestSet images, click “Set” and make a note of this Maximum value. You will be asked to enter this value into a pop-up window. The images in **TestSetHDM** will be mostly black with some faint light matix fibres and will open automatically for you to check. If you are happy with the images (you see some light matrix on a mostly black background) then click to proceed. If you think the images are too dark you will have the opportunity in Step 9 to increase the value.


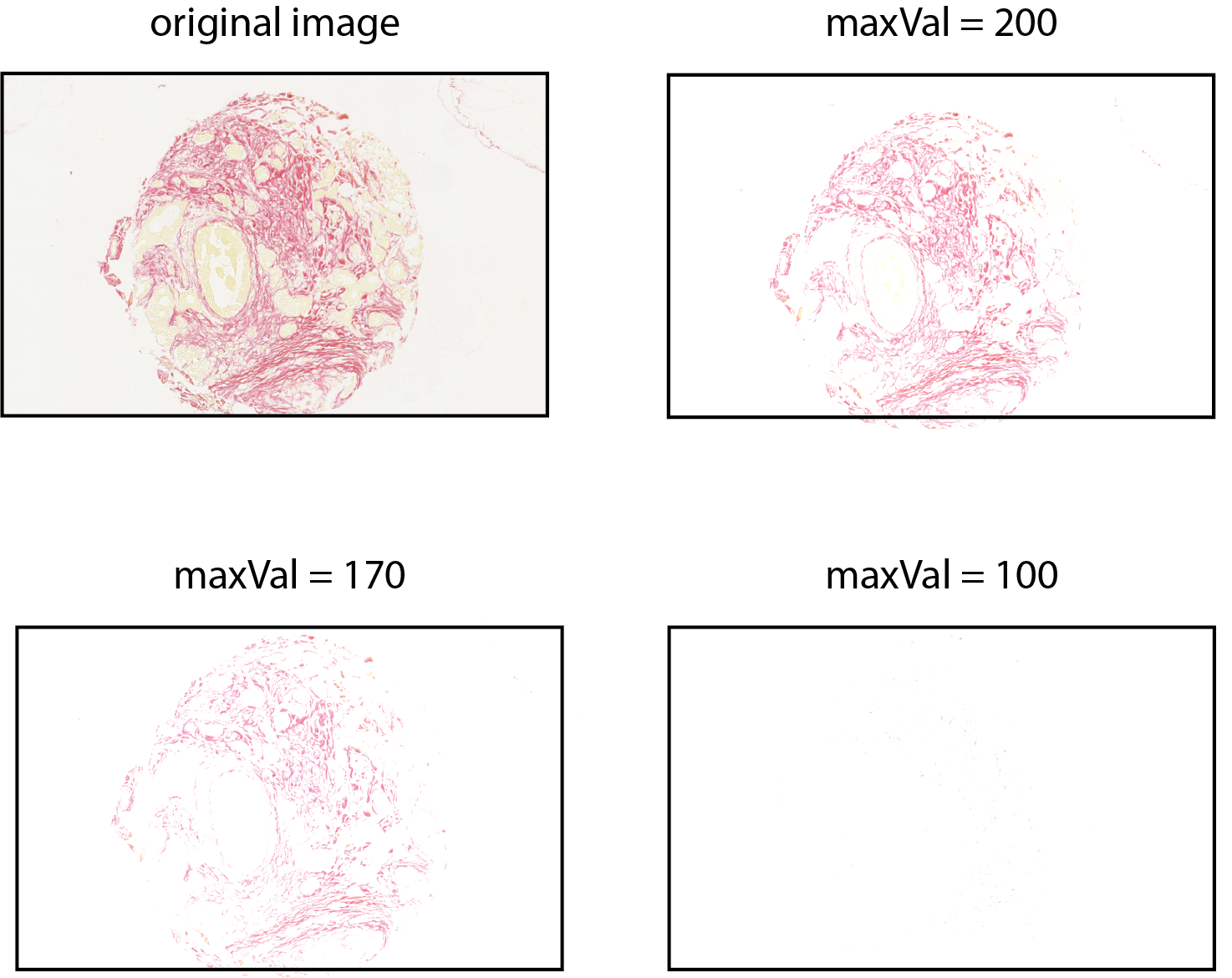


Figure 7: Choosing HDM threshold. Here a maxVal pf 200 (top right) is the most suitable value of maxval (a value of 170 would also be acceptable) as it removes the background and leaves only red collagen.

## Step 9: Recap of chosen parameters

A dialogue box will next open where you will have the opportunity to change any of the parameter values before proceeding to the next steps of analysis. You can check the output masks and HDM thresholded images in **tutorial/OriginalFiles/TestSet/TestSetMasks** and **tutorial/OriginalFiles/TestSet/TestSetHDM** respectively.

For the walkthrough, we suggest the values listed in Table 4.

Table 4: Suggested values for example

| **Parameter** | **Value** |
| --- | --- |
| Contrast Saturation | 0.35 |
| minLineWidth | 5 |
| maxLineWidth | 5 |
| minCurvatureWindow | 40 |
| maxCurvatureWindow | 40 |
| Minimum Branch Length | 5 |
| maximumDisplayValueHDM | 200 |

These parameter values will then be saved in a file called parameterFile.txt. You will be asked to specify where this file should be saved. We recommend saving this in the folder **twombli/programs**. This can be used in future to avoid having to choose the parameters again (see “Using a pre-existing parameter file” section).

## Step 10: Choosing directories

Having confirmed all chosen parameters on the test set, matrix analysis will now be performed on all the images in the Eligible folder. Users will be prompted to select an input folder (**Tutorial/OriginalFiles/Eligible**) and an output folder (**Tutorial/Masks**).

Next you will be asked to select an output folder for HDM images (**Tutorial/HDM**). This folder needs to be empty. If it is not, you will be prompted to empty it before continuing. TWOMBLI will output the thresholded images here together with the HDM results.csv file.

Finally, you will be asked to select the file **twombli/programs/AnaMorfProperties.xml**. We recommend leaving the file as it is. Users can alter program by altering lines of the xml file from true to false outputs.

## Step 11: Ridge detection

The ridge detection plugin will produce output masks of the images and save them in the chosen output folder

## Step 12: AnaMorf & Alignment

AnaMorf is an ImageJ plug-in originally designed for analysing the microscopic morphology of filamentous microbes. More information can be found [here](https://github.com/djpbarry/AnaMorf/wiki).

AnaMorf will now run producing a folder of the form **TWOMBLI/Tutorial/masks/AnaMorf…** Inside this folder is a copy of the properties.xml file and a csv file containing the results.

Global alignment is computed as the coherency of the image and uses the plugin OrientationJ. Further information on the metric can be found [here](https://pubs.rsc.org/en/content/articlepdf/2018/ra/c7ra12693j).

## Step 13: HDM

The csv file will have a column “% Black”. HDM is defined as $h=1-b$, where $b$ is % Black ie the proportion of pixels darker than the threshold MaximumDisplayHDM value. This HDM value is automatically computed in the main results file.

The main results file “TwombliResults.csv” in the TWOMBLI folder lists all the metrics for each image together. Use this for further analysis.

**The tutorial is now complete**

# Running TWOMBLI

Once comfortable with the tutorial, the user is encouraged to try TWOMBLI with their own data by copying their images into a subfolder of TWOMBLI and following the same procedure as described in steps 1-13, taking care to not use folders within the tutorial. **The user should create subfolders organised according as shown in Figure 1B**. File types should be consistent between images e.g. all Tiffs or all pngs to avoid artefacts from variations in image quality.

## Using a pre-existing parameter file

When you run the macro, the first thing you are asked is if you have a pre-existing parameter file you wish to load. You may have already decided on appropriate parameter values from previously running TWOMBLI. These will be saved in a file called **parameters.txt**. By clicking “Yes” you will be prompted to choose this parameter file. Uploading a pre-existing parameter file will enable you to skip immediately to step 9.

NB Gap analysis will be run if and only if in the parameter file, the variable “Minimum Gap Diameter” is set to a non-zero value.


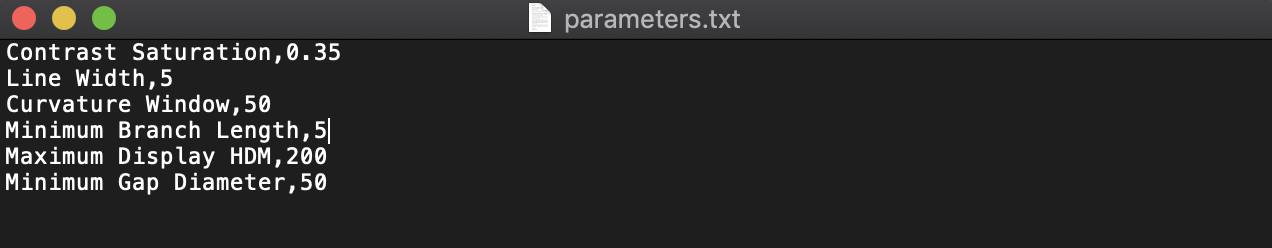


Figure 8: Example parameter file. Gap analysis will be run because Minimum Gap Diameter is not set to zero.

## Next steps

These matrix metrics quantify matrix patterns and can be used for quantitative analysis of the data. It is advisable to normalise the metrics as described [here](https://www.biorxiv.org/content/10.1101/867507v1). One such method is in survival analysis, when samples accompany clinical data. An introduction to survival analysis is given in <http://www.sthda.com/english/wiki/survival-analysis-basics>.

## Gap analysis

In step 7, TWOMBLI asks the user if they want to perform optional gap analysis. This algorithm is taken from the “Max Inscribed Circles function” in the “BIOP” plugin (<https://imagej.net/Max_Inscribed_Circles>) and aims to summarise the distribution of **large-medium size** gaps in the matrix structure by inscribing circles of decreasing size inside the gaps (Figure 6). It is not intended to analysis small gaps between close fibres. Gap analysis will then run as optional step 14.


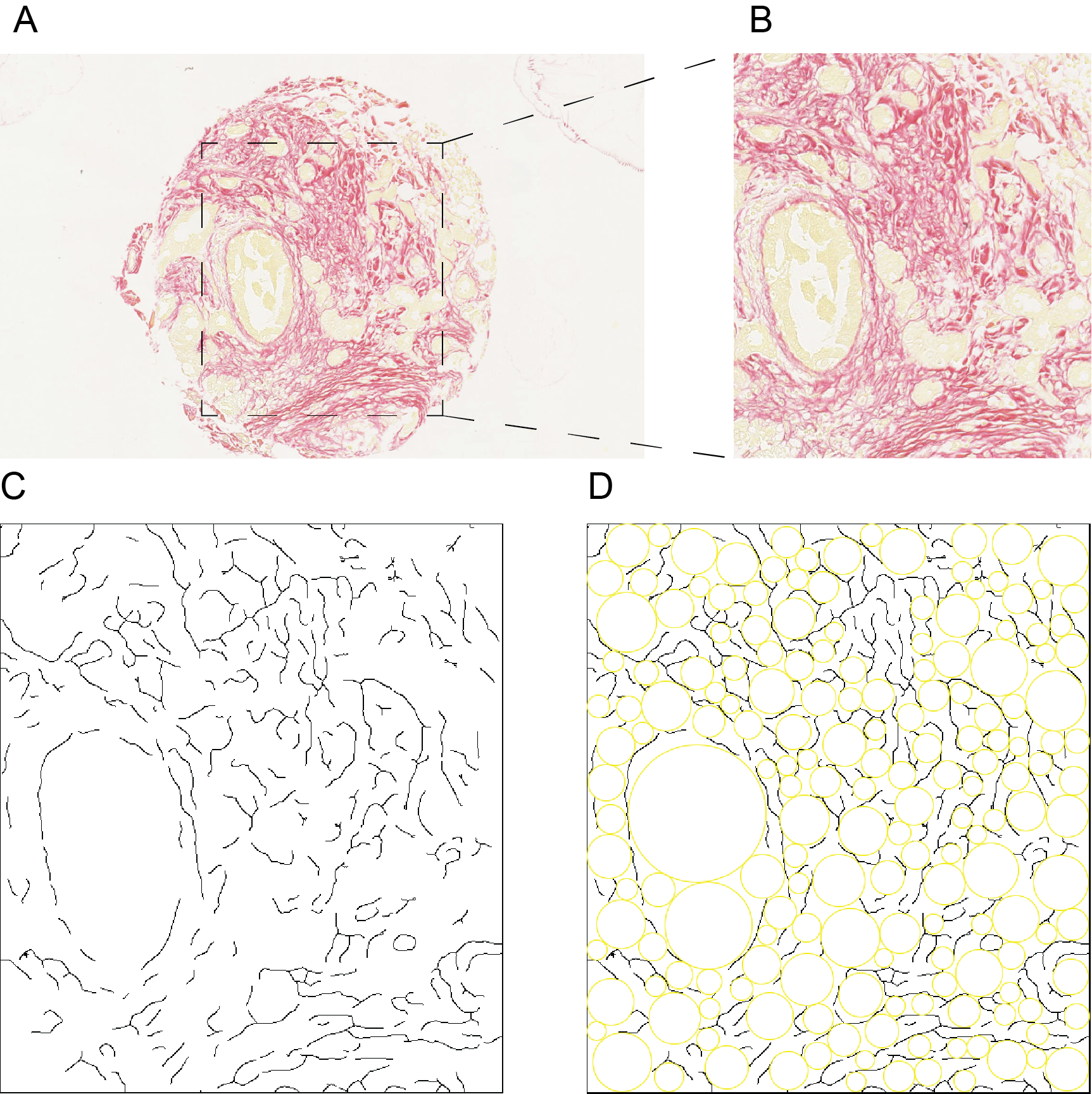


Figure 9: Gap analysis. (A) Samples with empty space eg a tumour core surrounded by a rectangle of background space need to be cropped down to exclude the background in order to perform gap analysis (B) Example tumour core cut down to exclude the background. (C) Mask corresponding to (B) on which gap analysis is performed. (D) Resulting gap analysis inscribes maximal circles between fibre curves which are automatically measured in a results file. Here minimum diameter = 20 pixels in an image of size ~500x450 pixels.

Only images without large amounts of background space are currently suitable for gap analysis. It may be necessary to crop images with background space beforehand if you want to perform gap analysis. If you select to do gap analysis (step 7), you will be asked to use the 'straight line' tool to decide the minimum diameter for the inscribed circles in the gap analysis (Figure , Figure 6). You should aim to find circle size that describes medium/large gaps. As a rough rule of thumb, the minimum gap diameter should be no smaller than $\frac{l}{30}$, where $l$ = (width of image + height of image)/2. This suggested value will automatically be suggested as the default value. The smaller the minimum diameter, the longer the gap analysis will take to complete and a very small can cause the system to crash. The output will be saved automatically in the directory containing the masks in a “GapAnalysis” subdirectory containing: **images** (Figure 6D), a **summary text file** (GapAnalysisSummary.txt) listing key summary statistics for each image, and **individual results** containing csv files analysing the circles filling the gaps in case the user wants to do more in-depth analysis.

## Selecting regions of interest

It may be the case that the images you want to analyse contain large regions that you wish to exclude, or only small areas of interest that you wish to include before running the pipeline. In this case, it is possible to pre-process the images using the separate macro **plugin->macro->run..->twombli/programs/croppingImages.ijm**, where it is possible to manually select either regions of interest or regions to exclude.


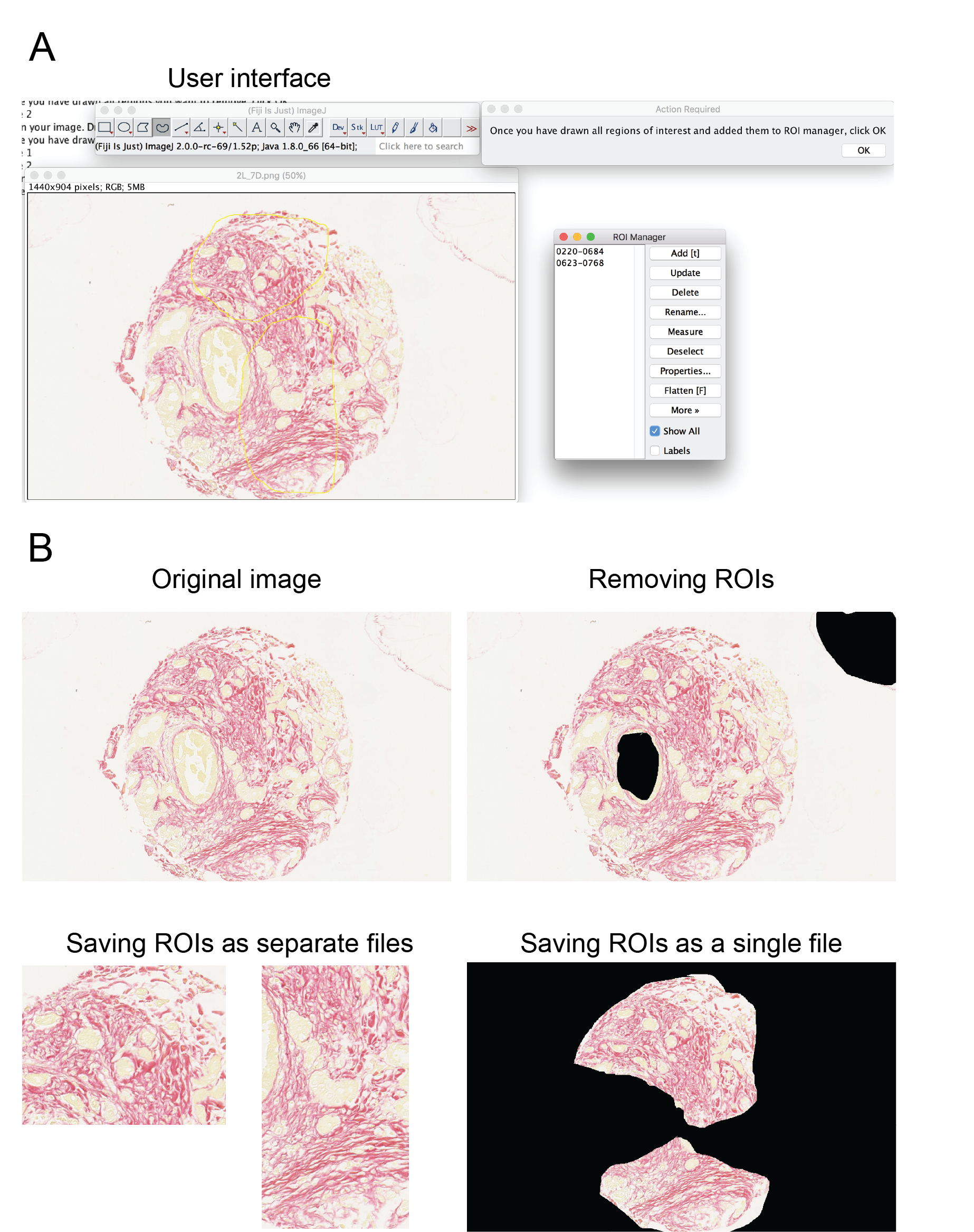


Figure 10: Selecting region of interests. Running the macro croppingImages.ijm before the TWOMBLI pipeline enables the user to select regions of interest (ROIs). (A) Example user interface. (B) The user has the option of saving the image with ROIs excluded (top right), with ROIs saved as separate files (bottom left) or as a single file (bottom right).

# Quickstart guide

1. Download Twombli from <https://github.com/wershofe/TWOMBLI>
2. Put suitable images you want to analyse in twombli/myProject/OriginalFiles/Eligible
3. Copy three of these images into twombli/myProject/OriginalFiles/TestSet/TestSetInput
4. Open Twombli_v1_0.ijm in twombli/programs and run
5. Follow the instructions in the log to choose parameters
6. Twombli will run on all your input images
7. Concatenate output results files

# Trouble shooting

TWOMBLI is running very slowly or running out of memory

**Images should not be bigger than approximately 5MB and 2000x2000 pixels, otherwise computer can run out of memory when computing Ridge Detection and AnaMorf steps. If your images are large, they should be binned (Image->Transform->Bin) or cropped.**

Masks are picking up other non-matrix components

**NB Images should be stained for matrix components so that matrix is the darkest stain in image. Corrections will need to be made if for example cell nuclei are stained darker, as otherwise the Ridge Detection will mistake them for matrix.**

**For any enquiries please contact erik.sahai@crick.ac.uk**
